# Molecular Targeting of Epidermal Growth Factor Receptor (EGFR) and Vascular Endothelial Growth Factor Receptor (VEGFR)

**DOI:** 10.3390/molecules26041076

**Published:** 2021-02-18

**Authors:** Nichole E. M. Kaufman, Simran Dhingra, Seetharama D. Jois, Maria da Graça H. Vicente

**Affiliations:** 1 Department of Chemistry, Louisiana State University, Baton Rouge, LA 70803, USA; nkaufm1@lsu.edu (N.E.M.K.); sdhing1@lsu.edu (S.D.); 2 School of Basic Pharmaceutical and Toxicological Sciences, College of Pharmacy, University of Louisiana at Monroe, Monroe, LA 71201, USA

**Keywords:** EGFR, VEGFR, TKI, tyrosine kinase, imaging, peptide, protein, overexpression, cancer

## Abstract

Epidermal growth factor receptor (EGFR) and vascular endothelial growth factor receptor (VEGFR) are two extensively studied membrane-bound receptor tyrosine kinase proteins that are frequently overexpressed in many cancers. As a result, these receptor families constitute attractive targets for imaging and therapeutic applications in the detection and treatment of cancer. This review explores the dynamic structure and structure-function relationships of these two growth factor receptors and their significance as it relates to theranostics of cancer, followed by some of the common inhibition modalities frequently employed to target EGFR and VEGFR, such as tyrosine kinase inhibitors (TKIs), antibodies, nanobodies, and peptides. A summary of the recent advances in molecular imaging techniques, including positron emission tomography (PET), single-photon emission computerized tomography (SPECT), computed tomography (CT), magnetic resonance imaging (MRI), and optical imaging (OI), and in particular, near-IR fluorescence imaging using tetrapyrrolic-based fluorophores, concludes this review.

## 1. Introduction

EGFR and VEGFR overexpression is frequently found in several types of tumors, including breast, lung, colon, and ovarian, and therefore it is a very attractive therapeutic and imaging target for cancer treatment research [1,2]. The complete detection of tumors, as well as tumor infiltrative areas and tumor metastasis, has an enormous impact on treatment planning, tumor response to treatment, and on overall treatment outcomes, and significantly increases the success of cancer treatment and the patient’s quality of life. Since molecular imaging allows noninvasive and repetitive imaging of dynamic processes, it has advantages over other conventional detection techniques that use biopsies and surgical procedures for cancer diagnosis, surgical guidance, and treatment monitoring. Therefore, molecular imaging plays a pivotal role in medicine, especially in the field of cancer diagnosis and treatment, as it provides accurate information regarding the stage and location of cancer by visualizing tumor properties at an early stage, evaluating therapeutic targets, and monitoring treatment outcomes. The most common molecular imaging modalities currently employed include positron emission tomography (PET), single-photon emission computerized tomography (SPECT), computed tomography (CT), magnetic resonance imaging (MRI), ultrasound imaging (US), and optical imaging (OI). In this review, we will summarize the contrast agents used in common molecular imaging modalities that specifically target the EGFR and VEGFR receptor families. We will focus on optical imaging methodologies, particularly the use of fluorescence imaging, as this technique provides superior sensitivity and resolution even at the subcellular level, giving real-time information on tumor cell properties and location. In addition, optical imaging techniques have become essential tools in the fundamental study of small animal models and in drug development.

The use of fluorescent dyes conjugated to biomolecules that are selective for over-expressed cell receptors, such as EGFR and VEGFR, has many advantages, as it enables visualization and detection of primary and metastatic lesions as well as fluorescence-guided surgery. Photostable dyes and nanoparticles with emissions within the near-IR region (600–900 nm) are particularly attractive since near-IR fluorescence displays low Raman scattering cross sections associated with the use of low energy excitation photons, larger Raman-free observation windows, and reduced absorption and fluorescence from other molecules. Furthermore, light penetration through human tissue typically increases with the wavelength. Therefore, conjugation of fluorescent dyes to antibodies or to peptide sequences directed at EGFR and VEGFR receptors, as well as to tyrosine kinase inhibitors (TKIs), is an effective strategy currently used in medical imaging and cancer treatment.

It is of great importance to enhance our understanding of the structure and structure-function relationship of the targeted receptors in order to design and create effective drug targets. To this end, we will first discuss the structure of the EGFR and VEGFR receptor tyrosine kinase (RTK) domains and their relevant function in kinase activity. We also review currently used imaging modalities for EGFR and VEGFR family proteins and currently used drugs for EGFR and VEGFR targeting and inhibition, with emphasis on low molecular weight molecules including peptides, peptoids, and TKIs.

### Receptor Tyrosine Kinases (RTKs)

The human protein kinase family is one of the largest gene families owing to their regulatory role in virtually every facet of cell biology [3]. Protein kinase enzymes catalyze the transphosphorylation of hydroxyl-containing substrates (typically proteins) via the conversion of adenosine triphosphate (ATP) to adenosine diphosphate (ADP) facilitated by a divalent cation (typically Mg^2+^) as depicted in the following substitution reaction centered on phosphorus:

MgATP^−^ + protein-O:H → protein-O:PO_3_^2−^ + MgADP + H^+^, where the γ-phosphoryl group (PO_3_^2−^), as opposed to a phosphate group (OPO_3_^2−^), is transferred from ATP to the substrate protein [4]. The transfer of the phosphoryl group depicts the initiation of intracellular signaling cascades to transduce the signal from the cell surface, through intracellular vesicles, to the nucleus in order to activate gene expression. Of the 518 identified protein kinase family members (2% of all human genes), 385 are serine/threonine protein kinases, 90 are protein-tyrosine kinases, and 43 are tyrosine-kinase like proteins. Of the 90 protein-tyrosine kinases, 58 are transmembrane receptors (containing extracellular, transmembrane, and intracellular domains), and 32 are intracellular non-receptors [4]. The human epidermal growth factor receptor (HER)/erythroblastic leukemia viral oncogene homolog (ErbB) and the vascular endothelial growth factor (VEGF) RTKs are among the most extensively studied cell signaling families in biology.

## 2. EGFR Family of Receptor Tyrosine Kinases (RTKs)

### 2.1. EGFR/ErbB/HER

The EGFR/ErbB/HER family of RTKs consists of four homologous transmembrane proteins that are ubiquitously expressed in epithelial, cardiac, neuronal and mesenchymal cells: (1) ErbB1, also referred to as epidermal growth factor receptor (EGFR) or human epidermal growth factor receptor 1 (HER1), (2) ErbB2/HER2/Neu, (3) ErbB3/HER3, and (4) ErbB4/HER4 [4]. The ErbB nomenclature is derived from the name of avian viral erythroblastosis oncogene to which EGFR is related. The epidermal growth factor (EGF) was first discovered in 1962 [5], followed by its receptor (EGFR) in 1978 [6], by the late Stanley Cohen was the first receptor to be characterized as a protein-tyrosine kinase. The ErbB/HER family exerts critical functions in cell physiology through canonical signaling pathways to regulate transcription, differentiation, proliferation, cell cycle progression, and apoptosis [2]. Noncanonical signaling pathways, generally induced by external factors (e.g., xenobiotics) or cellular and environmental stresses, are consequently activated in cancer cells conferring upon them a survival advantage and therapeutic resistance via induction of anti-apoptotic effects, aberrant angiogenesis, and initiation of metastatic growth, and promotion of tumor cell proliferation and growth [2].

### 2.2. Structure of EGFR

**Figure 1:**
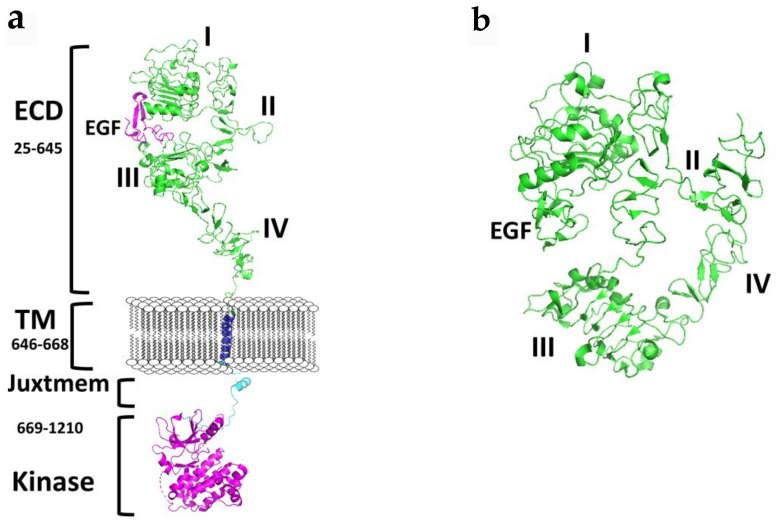
A schematic diagram of the structure EGFR. (**a**) Full-length EGFR showing extracellular domain (ECD) in an open conformation, transmembrane (TM), and the cytoplasmic kinase domain. Published crystal structures of EGFR were used to prepare the schematic. ECD (PDB ID: 3NJP); transmembrane (PDB ID:2KS1), Kinase domain with juxtamembrane domain (PDB ID: 3GOP). Notice the distance between domains II and IV in an open conformation. Dimerization arm II is open for dimerization interaction. (**b**) Structure of ECD of EGFR in the closed conformation (PDB ID: 1NQL). Domains IV is folded and interacts with II. In the closed conformation EGFR dimerization arm II is not available for dimerization. PyMol (Schrodinger LLC. OR) was used to generate the structures of EGFR. ECD, extracellular domain; eJM, extracellualr juxtamembrane; TMD, transmembrane domain; iJM, intracellular juxtamembrane; TKD, tyrosine kinase domain [11,14,15,16].

Each ErbB family member protein consists of a large glycosylated N-terminal extracellular domain (ECD) consisting of ~620 amino acids with a ligand-binding region [7]. ECD is subdivided into four domains (I–IV), where domains I and II have a beta-helix/solenoid structure, and domains II and IV are cysteine-rich domains that contain disulfide modules [4,8,9,10,11,12]. A single anchoring hydrophobic transmembrane domain (TMD) links the extracellular region to the cytoplasmic region via a juxtamembrane domain (JMD). Detailed structural analysis suggests two juxtamembrane regions, (a) an extracellular juxtamembrane, and (b) an intracellular cytosolic juxtamembrane. The extracellular juxtamembrane (eJM) has a short stretch of seven amino acids that links the TMD to the C-terminal domain of IV of ECD, while the intracellular juxtamembrane (iJM) separates the TMD from the kinase domain (Figure 1). The intracellular regions consist of nearly 540 amino acids that consist of a tyrosine kinase domain (TKD) and a carboxyterminal tail (CTT) of ~230 amino acids [4,13]. The C-terminal carboxy tail contains tyrosine amino acids that are important in phosphorylation and its structure is not yet well defined due to the flexibility of the structure.

### 2.3. Epidermal Growth Factor (EGF) Ligand Family

Eleven homologous growth factor ligands that activate ErbB receptors have been identified and can be categorized into three classes: (1) high-affinity ligands, (2) low-affinity ligands, and (3) neuregulins [17]. Structurally, all of the EGF-family members contain a central B-sheet hairpin surrounded by three highly conserved intramolecular disulfide bridges that form tightly coiled structural loops essential for receptor binding [17]. EGFR ligands are shown in Table 1.

It has been shown that seven of the EGF ligands can stimulate different downstream signaling effects: EGF, HB-EGF, and BTC promote receptor downregulation and a shorter signaling pulse, in contrast to TGF-a, AREG, EREG, and EPGN, which promote receptor recycling and a sustained signal [17].

### 2.4. Importance of EGFR Dimerization in Cell Signaling and Cancer

Receptor tyrosine kinases of the EGFR family control the cell growth and proliferation in normal cells, and any dysregulation of this process leads to cancer [18]. The binding of ligands to the extracellular domain initiates the signaling process that changes the conformation of ECD of EGFR, leading to homo- and heterodimers (with other members of EGFR), resulting in passing the signal from outside the cells to inside the cell by phosphorylation of the kinase domain. This process is facilitated by a change in the structure of EGFR that helps dimerization. Details of the extra- and intracellular domains structure have provided insights into the signal transduction process. However, most of the models proposed are based on crystal or NMR structures of individual domains as an elucidation of the entire intact protein receptor is not feasible. In the inactive state, EGFR is known to be in a “closed” conformation where there is a large gap between domains I and III, and domain IV is folded in such a way to “tether” to domain II [17] (Figure 1b). In this conformation, EGFR cannot form dimers as dimerization arm II and IV are not able to interact with other receptors. Upon binding of the ligand to ECD between domains I and III, a conformation change occurs, resulting in the opening of the tethered conformation where domains II and IV move far away from each other (Figure 1a). Domains II and IV of two EGFR molecules come into contact with one another, forming a dimer. It is known that among the EGFR family of receptors, EGFR, HER3, and HER4 have known ligands and are known to exist in an open and closed conformation. Although the overall structure of HER2 is similar to other EGFRs, HER2 does not have a known ligand and is always in the open conformation [19]. Thus, HER2 is the preferred dimerization partner for other EGFRs. Dimerization of ECD activates the intracellular kinase domains of the receptor resulting in autophosphorylation of a ~230 residue C-terminal tail and initiation of downstream signaling. Transmembrane domain and juxtamembrane domains are known to participate in the dimerization process that helps kinase activation [8,9].

On the cytoplasmic side, the kinase domains form an asymmetric dimer in which one kinase acts as an activator of the other dimer partner by transphosphorylation. For details of structural aspects of this process, readers can refer to reviews [13,16,20,21]. The C-lobe of one kinase (the ‘activator’) interacts with the N-lobe of another kinase (the “receiver”). The carboxyl-terminal tail of EGFR has autophosphorylation sites and acts as a binding region to SH2 domains. The tyrosine residues phosphorylated by EGF’s addition to cells include Y703, Y920, Y992, Y1045, Y1068, Y1086, Y1148, and Y1173. In addition to these autophosphorylated sites, there are also residues that are phosphorylated by other kinases, which interestingly appear downstream in the EGFR-activation cascade. For example, Y845 is phosphorylated by c-SRC [17], and T654 is phosphorylated by PKC [15]. EGFRs work together in a concerted manner to generate the signaling for cell growth. HER2 does not have a known ligand and hence is always ready to partner with EGFR/HER2/HER3. On the other hand, HER3 is known to have weak enzyme activity and hence does not participate in phosphorylation activity significantly. However, with EGFR, HER2, and HER4, HER3 dimerizes and participates in phosphorylation activity and signaling.

### 2.5. The Tyrosine Kinase Domain (TKD)

The homologous kinase domains of eukarytoic protein kinases (ePK) are responsible for catalytic activity with three distinct functions: (1) binding and proper orientation of the ATP phosphoryl donor-divalent cation complex, (2) binding and proper orientation of the phosphoryl acceptor protein substrate, and (3) transfer of the γ-phosphate from ATP to the hydroxyl or phenol containing residue (Ser, Thr or Tyr) of the protein substrate [22]. The ErbB/HER tyrosine kinase domain (TKD) is comprised of about 270 amino acids and, similar to all other protein kinases, has a small N-terminal lobe and a large C-terminal lobe [4]. Activation of the TKD is essential for cellular responses to ligand binding and is achieved via the juxtaposition of two monomer catalytic kinase domains to form an asymmetric dimer, where the donor TKD C-lobe abuts the acceptor TKD N-lobe. This leads to the trans-autophosphorylation of critical tyrosine residues in the C-terminal tails and triggers the signaling cascade [16]. 

**Figure 2:**
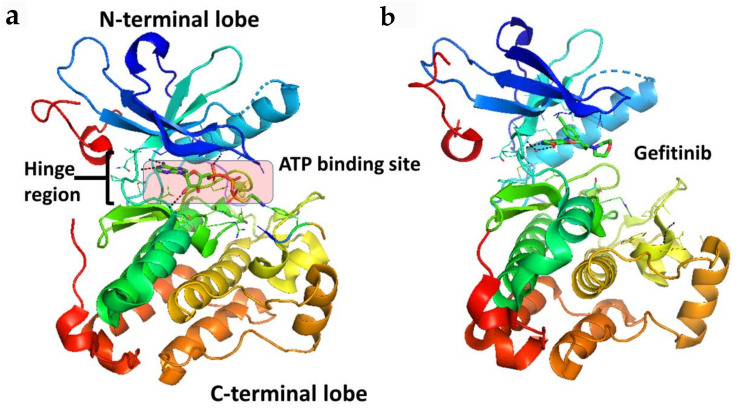
Crystal structure of the kinase domain of EGFR (**a**) with ATP binding site highlighted (PDB ID: 2GS6). TKIs of EGFR bind to EGFR in the ATP binding pocket, forming 1 to 3 hydrogen bonds to the hinge region. (**b**) EGFR kinase domain with gefitinib bound in the ATP binding pocket (PDB ID: 3UG2). PyMol (Schrodinger LLC. OR, New York, NY, USA) was used to generate the figure [33,34].

Taking a closer look at the ATP binding pocket, the space between the N- and C-terminal lobes (Figure 2) can be subdivided into the front cleft, gate area, and back cleft [4]. The gate area and back cleft comprise hydrophobic pocket II (HPII). The Sh2 residue is termed the gatekeeper due to its ability to control access to the back cleft. The front cleft contains the hinge residues and adenine-binding pocket, the flexible glycine-rich P-loop (named for its proximity to the phosphate groups of the ATP substrate), the catalytic loop motif (HRD(X)4N), and the portion connecting the hinge residues to the αD-helix [4]. The β1- and β2-strands of the N-terminal lobe dock with the ATP adenine moiety. The gate area consists of the β3-strand of the N-terminal lobe and the proximal section of the activation segment, including the DFG (Asp-Phe-Gly) motif. The back-cleft projects to the αC-helix to a portion of the αE-helix within the C-terminal lobe and to the N-terminal lobe segments β4- and β5-strands of the small lobe [4]. One of the hurdles in the development of protein kinase inhibitors is to increase selectivity to reduce unwanted side effects, a process that is facilitated by characterizing drug-kinase interactions [4,23,24]. 

Although ligand binding is needed for EGFR dimerization and activity in normal as well as cancer cells, ligand binding is not necessary in some cancer types. Mutation or overexpression of receptor proteins can lead to dimerization of receptors. Most of the proposed models of the mechanism of EGFR activation involve monomer to dimer transition. However, the existence of higher-order multimers was suggested in cells [25,26]. Using molecular modeling and single-molecule tracking studies it was shown that higher-order multimers are proposed [27,28,29]. Single-molecule tracking studies of EGFR in live cells shown that EGFR forms large clusters after activation [10,30,31,32].

## 3. VEGFR Family of Receptor Tyrosine Kinases (RTKs)

### 3.1. VEGFR

The vascular endothelial growth factor (VEGF) family of RTKs is comprised of three structurally similar transmembrane proteins: (1) VEGF receptor-1 (VEGFR-1), first called feline McDonough sarcoma (fms)-related tyrosine kinase-1 (Flt-1), (2) VEGFR-2, also known as kinase insert domain receptor (KDR) or the murine homolog fetal liver kinase-1 (Flk-1), and (3) VEGFR-3, or fms-related tyrosine kinase-4 (Flt-4) [1]. VEGFRs, initially reported to be expressed solely on endothelial cells, are found to be expressed on both endothelial and non-endothelial cells, including tumor cells^1^. These receptors, along with their ligands and co-receptors neuropilin-1 (NRP-1), neuropilin-2 (NRP-2), and the heparan sulfate proteoglycans (HSPGs) (glycoproteins containing glycosaminoglycan (GAG) chains(s)), mediate critical interactions in vasculogenesis, lymphangiogenesis, and angiogenesis [1]. Vasculogenesis is the de novo formation of blood vessels that typically occurs during embryonic development versus angiogenesis, which is the sprouting and splitting of pre-existing vasculator to form new blood vessels. The flux of vascular networks is mediated by local and systemic tissue demands, thus, angiogenesis aids in the repair of tissue (would healing) and in the general growth and maintenance of the organism [35]. Lymphangiogenesis, as the name suggests, involves the formation of new lymph vessels from pre-existing lymphatics.

As the diffusion limit of oxygen in mammalian tissue is around 100–200 μM, surrounding metabolically active tissue outside that range can become hypoxic without an adequate supply of blood and thus, oxygen [36]. Reflecting the extent to which oxygen can diffuse through tissue, in the absence of neo-vascularization, solid tumor growth is limited to about 0.2–2.0 mm in diameter lest it becomes hypoxic [36]. The complex and tightly regulated process of angiogenesis is dependent upon 30 pro- and 30 anti-angiogenic factors held in balance. A shift in the balance of these factors favoring the increase of pro-angiogenic factors, called an angiogenic switch, is critical in the malignant growth and progression of solid tumors [1].

### 3.2. Structure of VEGFR

**Figure 3:**
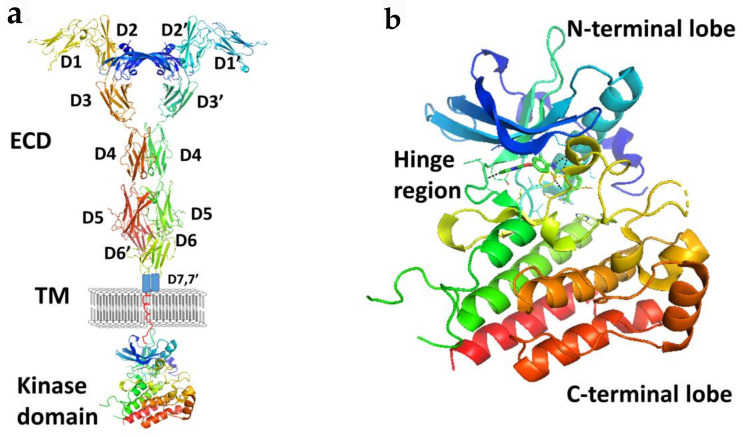
(**a**) A schematic representation of full length VEGFR-1 with VEGF-A. Extracellular domains D1 to D6 are from the crystal structure of VEGFR-1 (PDB ID: 5T89). D7 and TM are shown in schematic. Kinase domain was from the crystal structure of VEGF (PDB ID: 3VHE). (**b**) Kinase domain of VEGF (PDB ID: 3VHE) showing hinge region and N and C-terminal lobes. PyMol (Schrodinger LLC. OR) was used to generate the figure [37,38].

Members of the VEGFR family typically consist of an extracellular ligand-binding domain (ECD) with a seven immunoglobulin (Ig)-like motif (with connecting linkers), a single transmembrane domain, and an intracellular region containing a juxtamembrane domain (JMD) and a tyrosine kinase domain (TKD) split by a kinase insert and a carboxyl terminus (Figure 3). VEGFRs may induce intracellular signal transduction as homo- and/or heterodimers with different phosphorylation patterns occuring in heterodimers compared to homodimers.

VEGFRs are activated upon ligand-mediated dimerization. It is suggested that ligand-induced receptor dimers are stabilized by both ligand-receptor as well as homotypic receptor-receptor interactions. Ligand binding in the extracelullar domain induces transmembrane signaling that results in cross-phosphorylation in the intracellular kinase domains. It is interesting to note that while VEGFR1 and VEGFR2 share 44% homology, they differ vastly with respect to their structure and function. For example VEGF-A binds to VEGFR1 and VEGFR2 but does so via different domains (Ig domain 2 and 3, respectively) of the VEGF-A ligand. Thus, mutations that disrupt the binding of VEGF-A with VEGFR1 may not necessarily affect the binding with VEGFR2 and vice versa.

The neuropilins (NRPs) are 120–140 kDa transmembrane non-protein-tyrosine kinase glycoproteins that act as co-receptors for both the semaphorin family and the VEGF family. Neuropilins contain large (~250 kDa) transmembrane plexins that transduce semaphorin signaling as co-receptors with VEGFR1, VEGFR2, and VEGFR3 that transduce VEGF family signaling. Neuropilins also function as receptors for VEGF isoforms independently of VEGFR1, VEGFR2, or VEGFR3. NRPs are pleiotropic receptors, and therefore, other molecules may interfere with the signaling of the NRP-VEGF receptor complexes. They consist of a large extracellular domain, a transmembrane segment, and an intracellular domain composed of approximately ~40 amino acid residues, which are relatively short to perform a catalytic function. Therefore, it is possible that the intracellular domain serves as a docking site for downstream molecules, either alone or in conjunction with co-receptors.

### 3.3. Vascular Endothelial Growth Factor (VEGF) Ligand Family

There are seven VEGF family members that have been identified thus far that specifically interact with VEGFRs 1–3, neuropilin-1 and -2, and heparin sulfate proteoglycan co-receptors (see Table 2): vascular endothelial growth factor A (VEGF-A), VEGF-B, VEGF-C, VEGF-D, viral homolog VEGF-E, snake venom homolog VEGF-F, and placenta growth factor (PlGF). Generally, these ligands are structurally similar disulfide-linked homodimeric glycoproteins that can undergo alternative splicing to express biologically relevant isoforms, namely VEGF-A and VEGF-B (i.e., VEGF-A121, VEGF-A145, VEGF-A165, VEGF-A189, VEGF-A206, and VEGF-B167 and VEGF-B186).

Produced predominantly by endothelial, stromal, and hematopoietic cells, VEGF glycoproteins are secreted upon stimulation by growth factor [i.e., transforming growth factor β (TGF-β), interleukins, and platelet-derived growth factors (PDGFs)]. Originally identified as vascular permeability factor (VPF), the ~35 kDa homodimeric glycoprotein VEGF-A is the most potent/prevalent angiogenic growth factor/protein influencing angiogenesis.

## 4. Targeting EGFR and VEGFR with Different Inhibition Modalities

### 4.1. Tyrosine Kinase Inhibitors (TKIs)

**Figure 4:**
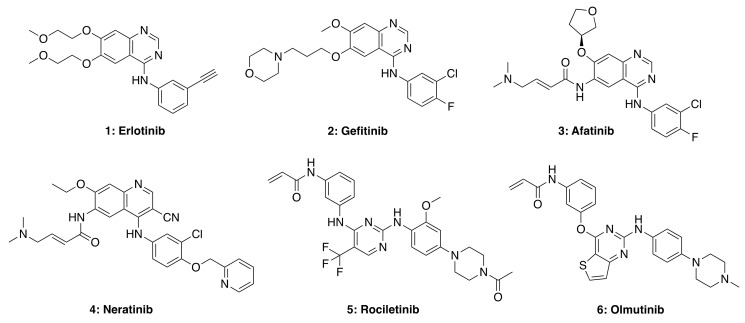
Representative structures of first (**1** and **2**), second (**3** and **4**), third (**5**), and fourth (**6**) generation EGFR TKIs. Structures were generated using Chemdraw based on structures of TKIs available in selleckchem.com.

Many biologically and pharmacologically significant drugs are heterocyclic compounds that are also highly relevant in medicinal chemistry. In particular, quinazoline-based compounds are an important class of heterocyclic pharmacophores that have been shown to possess a wide range of biological activities, including analgesic, anti-inflammatory, anti-hypertensive, anti-cancer, antimicrobial, antibacterial, antifungal, anti-HIV, anti-malarial, and anti-viral properties [40]. The quinazoline scaffold is considered a privileged structure and serves as the framework core for several small-molecule tyrosine kinase inhibitors (TKIs), as shown in Figure 4. The quinazoline structure itself is a mancude heterobicyclic planar molecule containing a benzene ring fused to pyrimidine via two adjacent carbon atoms (a.k.a., 1,3-diazanaphthalene). The total synthesis and antitumor activity of 4-aminoquinazolines have been recently reviewed [41].

Small molecule TKIs function by preventing phosphorylation through the obstruction of protein kinase activity via (1) competitive binding with ATP, (2) competitive binding with the substrate, (3) competitive binding with both ATP and substrate, or (4) allosteric inhibition (e.g., affecting protein conformational change) [42,43]. TKIs are typically utilized in targeted therapy, selectively identifying and attacking cells that express mutations or overexpress tyrosine kinase receptors. First-generation TKIs, such as Erlotinib (**1**) and Gefitinib (**2**), are reversible inhibitors that compete for binding to the kinase domain with endogenous ATP, therefore preventing kinase phosphorylation, blocking downstream signaling and cell regulation. Both these drugs are FDA-approved for the treatment of non-small cell lung carcinoma (NSCLC). However, due to acquired patient resistance to these drugs, second-, third- and fourth-generation TKIs were developed. Afatinib (**3**) and Neratinib (**4**) are second-generation TKIs that bind irreversibly to cysteine residues in the kinase domain; such stronger interaction with the ATP binding site can also cause side-effects in patients treated with these second-generation TKIs, including skin rashes and diarrhea. Third- and fourth-generation TKIs, such as rociletinib (**5**) and olmutinib (**6**), are selective against certain mutants (e.g., EGFR T790M, and C797S, respectively) but show limited wild-type EGFR inhibition. Therefore these drugs do not show as many side-effects observed in first- and second-generation TKIs. Nevertheless, most third-generation TKIs also develop resistance in patients due to receptor alterations and mutations, while fourth-generation TKIs are currently being investigated for C797S mutation-acquired resistance.

Improved tumor delivery of TKIs by using various formulations and nanoparticle delivery systems have been reported. For example, cyclodextrin-modified PLGA nanoparticles loaded with erlotinib were shown to improve the therapeutic efficacy against non-small cell lung cancer (NSCLC) [44]. Chitosan-based nanoparticles increased the biocompatibility and biodegradability of erlotinib and resulted in enhanced cytotoxicity against A549 lung cancer cells [45]. Superparamagnetic iron oxide nanoparticles conjugated with erlotinib [46] and gold-based nanoparticles bearing erlotinib and doxorubicin [47] have also been reported. Lipid-based nanoparticles have been used to deliver erlotinib [48] and afatinib [49] and are shown to have enhanced therapeutic efficacy against NSCLC. Similarly, hyaluronic acid-based nanoparticles have been explored for the delivery of erlotinib [50] and afatinib [51] to target tumors.

### 4.2. EGFR Antibodies, Nanobodies, and Small Peptide Ligands

As mentioned in the introduction, EGFR alteration such as mutation, overexpression, and gene amplification is found in many cancer types such as lung, breast, colon, and rectum as well as head and neck cancer [52,53,54]. Antibodies have been targeted to EGFR to reduce the signaling for cancer cell growth and are used as therapeutic agents [54]. Antibodies trastuzumab and pertuzumab are used in HER2-related breast cancer. Nearly 80% of colorectal tumors overexpress EGFR, and hence EGFR is one of the major targets in colorectal cancer (CRC). Antibodies cetuximab and panitumumab are used as therapeutic agents for metastatic colorectal cancer (mCRC). Approximately 40% of patients develop metastatic disease [55]. However, only 10% of advanced metastatic colorectal cancers respond to EGFR antibody therapy [56,57]. Cetuximab and panitumumab both bind to ECD domain III of EGFR and prevent the binding of EGFR ligands resulting in locking the EGFR in the “closed” or autoinhibitory conformation. This leads to the prevention of dimerization of EGFR and hence downstream signaling. It is postulated that the antibody-receptor complex is then internalized and either degraded or recycled, and this turnover is controlled by the ubiquitin-proteasome system [58,59,60,61].

**Figure 5:**
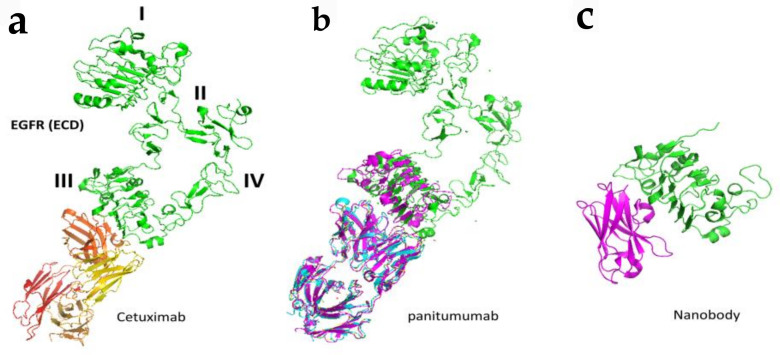
Binding modes of antibodies and nanobodies to EGFR extracellular domain. (**a**) Fab region of antibody cetuximab (red and yellow) bound to EGFR (green) domain III of ECD (PDB ID: 1YY9). (**b**) Panitumumab bound to EGFR ECD at domain III (PDB ID: 5SX4). (**c**) Crystal structure of nanobody bound to domain III of EGFR ECD. Only domain III is shown (PDB ID: 4KRl). PyMol (Schrodinger LLC. OR) was used to generate the figure [72,73].

Detailed binding analysis of cetuximab and panitumumab revealed that both cetuximab and panitumumab compete with EGF for its binding site to bind to EGFR (Figure 5). However, both antibodies have slightly different binding sites, and the binding epitope on EGFR is in proximity. In terms of binding affinity, both have different affinities to bind to EGFR, with panitumumab binding having an 8-fold higher affinity than cetuximab. Panitumumab binds near residues P349, P362 D355, F412, and I438 on EGFR, whereas cetuximab binds near residues Q384, Q408, H409, K443, K465, I467, and S468, as well as F352, D355, and P387. Panitumumab’s binding epitope overlaps with the EGF binding site in two locations (D355 and K443), whereas cetuximab overlaps with EGF’s binding site in five locations (D355, Q408, H409, K443, and S468) [62,63]. In addition to inhibiting the formation of open conformation, these antibodies induce antibody-dependent cellular cytotoxicity in vivo by recruiting immune cells to tumor cells (Figure 5) [64,65].

Resistance is a common mechanism after treatment with cancer therapeutic agents in most cancers. However, in colorectal cancer, resistance to antibody treatment is not common but found in few cases. The development of resistance was due to acquired mutations in the antibody binding region of EGFR [66,67]. It was found that resistance develops due to the substitution of amino acid arginine at S468 (S468/492R) in vitro as well as in clinical samples [68].

In cetuximab-resistant colorectal tumors, the following somatic mutations are observed that map to the cetuximab epitope on EGFR: G441/465R, G441/465E, and K443/467T. Other mutations, including S440/464L and I467/491M, are also seen in colorectal cancer cell lines. Substitutions at G441/465 and S440/464 have also been observed in panitumumab-resistant colorectal tumors [59,61,66,67,68,69,70]. However, another antibody, necitumumab, is known to bind to EGFR with cetuximab/panitumumab resistance EGFR [24]. Crystal structure of necitumumab Fab (Fab11F8) in complex with isolated domain III from EGFR (sEGFRd3) with S468R substitution (sEGFRd3-S468R) indicated that R substitution does not affect the binding of antibody necitumumab. Antibody matuzumab binds to domain III of ECD of EGFR, but the action of the mechanism is different from cetuximab and panitumumab. It binds to a different epitope on domain III compared to cetuximab and inhibits EGFR by preventing the activating conformational transition [71]. Structural analysis studies of matuzumab: EGFR indicate that the binding of matuzumab to domain III sterically blocks the conformational changes of domain III of EGFR, decreasing the ligand affinity for binding to EGFR. Therefore, it does not competitively bind with other antibodies, such as cetuximab.

Antibodies are effective in binding to specific target receptor molecules, however, they have limitations in terms of delivery, tumor penetration, and production because of their size [73]. The antigen-recognition region in antibodies consists of the variable regions of both heavy (VH) and light chains (VL). An antibody fragment consisting of a single monomeric variable region fragment is capable of binding selectively to an antigen and is called nanobodies. This single Ig domain is stable and can be generated rapidly and cheaply with simple expression systems [74] and are called VHH domains. These VHH domains are being developed for a range of research applications [75]. For therapeutic use, VHH domains (monomeric or multivalent) can be modified to extend serum half-life and/or functionality [76]. Such nanobodies are investigated for EGFR binding [77,78,79,80] (Figure 5).

**Figure 6:**
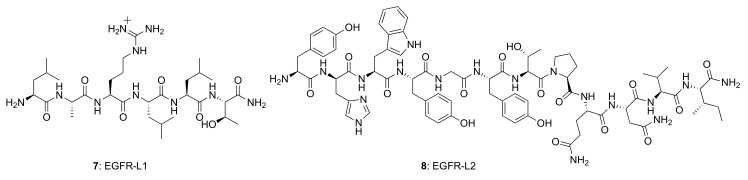
Structures of EGFR-targeting peptides, EGFR-L1 and EGFR-L2.

**Figure 7:**
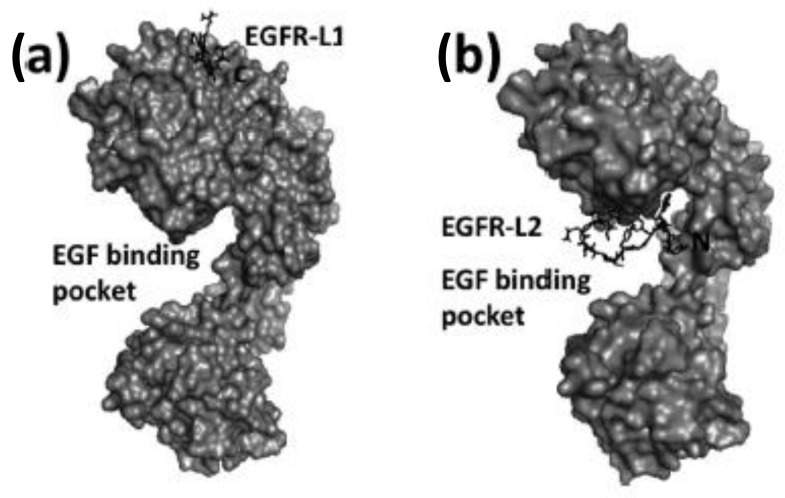
Structures of peptide ligands on their binding sites: (**a**) EGFR-L1 in domain I, and (**b**) EGFR-L2 in the EGF binding pocket (PDB ID: 1NQL) proposed based on docking studies. Peptides are shown as sticks, and EGFR is shown in surface representation. This figure was reprinted with permission from Reference [83].

Several small peptides have been proposed that bind to the EGFR extracellular domain, inspired by the natural ligands and identified from the combinatorial screening of various peptide libraries. Such peptides benefit from their easy and low-cost synthesis, high specificity, and high flexibility regarding their sequence, derivatization, and conjugation possibilities. In particular, two EGFR small peptide ligands, LARLLT (**7**) designated EGFR-L1 and YHWYGYTPQNVI (**8**) designated EGFR-L2, have been intensely investigated due to their low immunogenicity, ease of conjugation to various molecules, and superior EGFR-targeting ability [81,82] (Figure 6). The EGFR-L1 peptide was selected from the computational screening of a large peptide library and shown to target EGFR in vitro (using H1299 cells overexpressing EGFR) and in vivo (using H1299 tumor-bearing mice). This peptide was shown to bind specifically to the domain I on the ECD of the EGFR protein, as shown in Figure 7. On the other hand, EGFR-L2 was selected from screening a phage display peptide library and also shown to specifically bind to the EGF binding pocked of EGFR, in vitro (using SMMC-7721 cells) and in vivo (using SMMC-7721 tumor-bearing mice).

EGFR-L1 peptide was known to bind domain I of EGFR whereas, EGFR-L2 was known to bind to EGF binding pocket [81,82]. We have used docking methods to investigate the binding of EGFR-L1 and L2 peptides. The binding of these peptides was calculated based on low energy docked structure. The proposed models of binding of these peptides to EGFR ECD are shown in Figure 7 [83]. Docking studies were further confirmed by competitive binding studies and surface plasmon resonance (SPR) analysis. The peptide EGFR-L1 exhibited a β-turn structure upon binding to EGFR. Based on this β-turn structure, we further modified the peptide for its conformational as well as chemical stability and designed a cyclic version of the peptide [84]. The cyclic version of the peptide exhibited a higher affinity to bind to EGFR compared to the linear version of the same peptide.

Recently a derivative of EGFR-L2, bearing a glutamic acid residue in place of glutamine, showed enhanced cellular uptake in EGFR-overexpressing cells as a result of stronger binding to the EGF binding site [85].

Other peptides targeting different domains of EGFR were studied either for inhibition of dimerization or downregulation of phosphorylation. As explained above, EGFR extracellular domain II is important in stabilizing the dimerization. Peptides and small molecules have been targeted to inhibit the domain II dimerization arm. Mizuguchi et al. have described a cyclic peptide with conformational constraints such as a β-turn that inhibits EGFR dimers. The structure–activity relationship of differently designed peptides indicated that retro-inverso sequences of the dimerization arm exhibited antiproliferative activity in A431 cells and inhibited the dimerization and phosphorylation of EGFR [86]. Based on the peptides designed from the dimerization arm, bivalent ligands with optimized linkers (connected by poly(L-proline) or poly[(glycine)4(L-serine)]) were used to target EGFR. These bivalent ligands were proposed to bind to two EGFRs simultaneously and inhibit phosphorylation of the EGFR kinase domain. These bivalent ligands exhibited increased inhibition of phosphorylation compared to the monomeric peptide [87]. Peptides developed from the dimerization arm were also used to target adeno-associated virus (AAV) to EGFR expressing cells [88].

Apart from the extracellular domain, the juxtamembrane region of EGFR (Figure 1) is targeted by peptides to reduce the EGFR signaling for cell growth in cancer cells. A linear peptide was designed first and was shown to inhibit EGFRs activation [89]. Later, a stapled peptide was designed to stabilize the conformation and in vivo stability of the helical peptide. The stapled peptide was shown to be more effective in vivo in cancer models to reduce tumor growth [90]. Furthermore, the substrate binding site of the kinase was also targeted by peptides to EGFR resistant cancer cell lines. Tavakoli et al. [91] designed a library of peptides based on computation work to target mutant EGFR kinase. Such a library of peptides will be helpful to design conjugates of fluorophores to target EGFR. Peptides were also designed to inhibit the interaction of EGFR with EGF. Based on the binding surface of EGFR with EGF, Foy et al. have designed peptides for therapeutic purposes as well as for vaccine development for cancer [92].

Peptidomimetics have been designed and investigated that effectively target the EGFR ECD inhibiting protein-protein interactions and phosphorylation of the kinase domain, therefore modulating the signal for cell growth [93]. Based on the dimerization site of domain IV of extracellular domains of EGFR peptidomimetics that target EGFR dimerization inhibition were designed by our group. The designed peptidomimetics target HER2 protein rather than EGFR. However, the peptidomimetics designed to inhibit both EGFR:HER2 and HER2:HER3 dimerization [94,95,96].

## 5. Targeting EGFR and VEGFR for Molecular Imaging

Molecular imaging plays a pivotal role in medicine, especially in the field of cancer diagnosis and treatment, as it accurately provides information regarding the stage and location of cancer by visualizing the tumor properties, evaluating therapeutic targets, and monitoring treatment and outcomes [97]. Modern molecular imaging modalities currently in use for the detection of EGFR- and VEGFR-overexpressed cancers include PET, SPECT, CT, MRI, US, and OI (see Table 3). Molecular imaging provides information on receptor status not only at the tumor site but also at the sites where the tumor reaches the vital organs and biopsy is not possible. It is an important tool for early screening and diagnosis of disease, for focused and personalized patient therapy, and for measuring the effectiveness of therapy so that adjustments can be made and treatment based on the patient’s response can be designed. Moreover, it can also prevent false positive or false negative results due to the heterogeneity of receptor expression that occurs in individual biopsy specimens [98].

In order to develop an effective therapeutic agent, the targeting moiety or ligand must be attached with the appropriate labeling agent depending upon the imaging modality employed. For instance, PET and SPECT imaging modalities utilize radionuclides; optical imaging requires fluorescent dyes, quantum dots (QDs), or nanoparticles; MRI uses paramagnetic or superparamagnetic metal oxides; and ultrasound molecular imaging requires microbubbles, which are microspheres filled with perfluorobutane gas; CT utilizes emulsions, liposomes, lipoproteins and polymeric nanoparticles for imaging. The purpose of these imaging techniques is to provide real-time visuals of the receptors, over-expressed on tumor cells, clearly differentiating them from the normal cells to aid in designing novel therapeutics against cancer. Therefore, the high affinity of the targeting moiety must not be compromised even after binding with the labeling agent in order to obtain excellent resolution and quantitative diagnostic accuracy. Moreover, the process of contrast agent production should be cost-effective without compromising its quality.

### 5.1. Antibody-Based EGFR Imaging Agents

As discussed above, antibodies, nanobodies, and peptides have been targeted at EGFR for therapeutic purposes. Some of these have limitations in terms of the development of resistance and new antibodies that overcome the resistance have been designed using epitope mapping. Since these antibody molecules have high affinity and specificity to bind to EGFR (wild and mutated), antibodies can be labeled with imaging agents as indicated in Table 3. Apart from antibodies to EGFR, EGF-based ligand imaging probes are also designed. Some of these agents have been reviewed by Chen et al. [124]. Antibody-based imaging agents can be classified as whole antibody imaging agents, nanobodies, Fab fragments, and affibodies.

EGF, a natural ligand for EGFR, is labeled with gallium-68 using DOTA chelating agent for PET imaging. Li et al. have used fluorine-18 to label EGF. However, these agents have limitations in terms of imaging due to rapid clearance from the body. Since antibodies have slow clearance from the body, cetuximab (65 to 95 h half-life) was labeled with ^111^In or ^99m^Tc for SPECT imaging (Table 3). Apart from radiolabeling, fluorescent-based imaging agents were also developed. Cetuximab was labeled with commercially-available IRDye800CW, a cyanine-based near-IR dye. These fluorescent imaging agents are suitable for analysis of tissue layers at the cellular level in vitro, in vivo animal models, as well as in patient tissue samples [125].

Molecular imaging agents developed with full-length monoclonal antibodies have several limitations, including relatively high molecular weight and size, which limits their penetration into tissues in the tumor area. Furthermore, they tend to produce high imaging background and poor imaging quality. To overcome these limitations, Fab, the antigen-binding fragment of an antibody, is used. These fragments have high specificity of the whole IgG but have superior pharmacokinetic and nonimmunogenic properties compared with antibodies. These Fab fragments can be linked to radionucleotides or to fluorescent imaging probes, making them useful imaging agents with the ability to target certain proteins in cells and tissues. Cetuximab Fab was labeled with ^111^In for imaging purposes using this technology [126,127].

Another class of molecules that are derived from antibodies are designated affibodies, which are mimics of antibodies. The general structure of these molecules consists of three-helical bundles that are derived from the natural receptor for the Fc-portion of IgG. The amino acids in the helical bundles of the two helices of the three can be randomized and large libraries of affibody structures can be generated. Among the several generated sequence of structures, potential binding affibody to a particular receptor can be chosen. Since these molecules are relatively small proteins (~7 kDa), the designed peptides can be made to obtain suitable physicochemical properties for solubility and route of administration. Moreover, these small proteins can be conjugated to different imaging probes [128,129]. Zhao et al. [130] conjugated EGFR-targeting affibody to Ac-Cys label and Alexa680 (Cys-ZEGFR:1907). Affibodies have also been labeled with a fluorine-18 probe and to an iron oxide nanoparticle for PET, optical, and MRI imaging [131,132,133,134,135].

### 5.2. Antibody-Based Imaging Agents for VEGFR

As mentioned above, VEGF and its receptors play a critical role in the progression of metastasis in many cancers. High expression of VEGFs and their receptors were found in tumor tissue samples [136]. VEGF is also a well-known therapeutic target in CRC. Monoclonal antibodies that are used in the treatment of angiogenesis can be grouped into two categories, those that (a) bind to VEGF and inhibit VEGF receptor interactions, and (b) bind to VEGF receptors and activate the immune response. There is another class of molecule, VEGF-Trap, a fusion protein that consists of VEGFR-1 and VEGFR-2 binding domains and Fc region of IgG1 antibody. This fusion protein binds to VEGF-A, VEGF-B, inhibiting the activation of VEGFR-1 and 2 and hence angiogenesis [137,138]. Antibody bevacizumab reduces angiogenesis by blocking VEGF-A and is used as a therapeutic agent for treating CRC [139,140,141,142]. Ranibizumab is another antibody that has a high affinity for VEGF-A isoforms [143]. 89Zr-Df-Ranibizumab was used as VEGF-PET imaging agent in different types of cancers to analyze angiogenesis changes following treatment of TKIs [144]. Luo et al. have used 64Cu-NOTA-RamAb, a ramucirumab-based PET imaging agent for mapping VEGF-2 expression in vivo [145]. VEGF-trap (Zif-aflibercept) is also used for the treatment of CRC. Zr-89-labeled bevacizumab was used as an imaging agent for visualizing VEGF expression. However, because of the limitations in PK properties of Zr-89 labeled antibody, the agent could not be efficiently used for CRC imaging [146]. Zhang et al. developed a 64Cu-based imaging agent using bevacizumab. Tetrazine was conjugated with bevacizumab and pre-targeted immune-PET near-IR fluorescence was used for imaging VEGF expression. They showed that using biorthoganol chemistry VEGF-overexpressing CRC tumors could be imaged using pre-targeted immune-PET and near-IR fluorescence imaging. The conjugate was tumor-specific, and tumor to background contrast of the image could be achieved using this technique [147]. VEGF was also used for targeting a liposome with imaging agents to tumors. Zanganeh et al. developed an agent for near-infrared fluorescence diffuse optical tomography (FDOT) using indocyanine green (ICG) and a single-chain version of VEGF (scVEGF), the conjugate scVEGF-Lip/ICG [148]. Higher tumor accumulation of the tracer and longer clearance (t_1/2_ ~ 90 min) was found compared to the ICG-based tracer alone.

### 5.3. Fluorophore-Peptide Conjugates for Targeting EGFR and VEGFR

**Figure 8:**
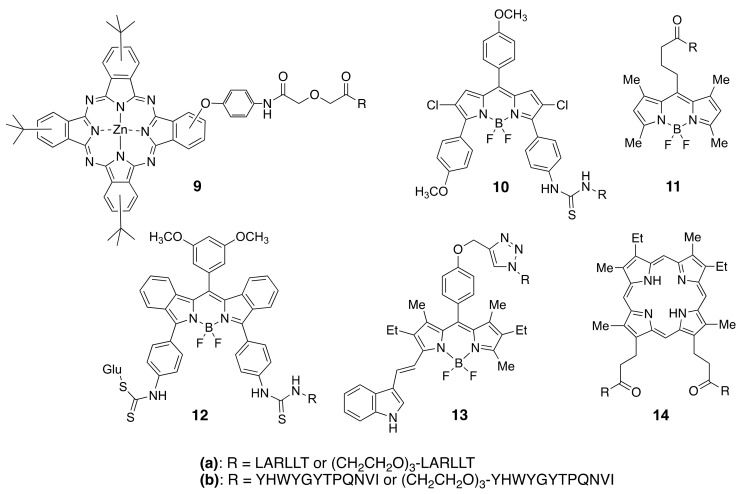
Structures of fluorophores conjugated to EGFR-L1 and EGFR-L2 peptides.

Among the different types of small-molecule ligands with the ability to bind to tyrosine kinases, it is peptides, peptomimetics, and TKIs that have been the most used for conjugation to fluorophores. Our group has selected two peptide sequences, EGFR-L1, and EGFR-L2 (Figure 6), which have been shown to bind to the ECD of EGFR with high affinity for conjugation with phthalocyanine [83], porphyrin [149], and BODIPY fluorophores [150,151,152,153]. Such fluorophore-peptide conjugates linked via a short or a longer tripegylated linker, as shown in Figure 8, showed enhanced cell-targeting ability compared with the unconjugated fluorophore. Surface plasmon resonance (SPR) and computational (Autodock) studies showed that the conjugates were able to bind to the known binding sites for the peptides (in domain I for LARLLT and within the EGF binding pocket for YHWYGYTPQNVI, as shown in Figure 7), and that the fluorophore further stabilized the structure of the conjugates in the peptide-binding sites due to additional interactions with hydrophobic residues on the EGFR protein. In the case of phthalocyanine (Pc) conjugates **9**, the most promising were found to be **9a**, in part due to their enhanced solubility and EGFR-targeting ability compared with **9b**, bearing the more hydrophobic peptide. The Pc conjugates were evaluated in vitro using several cell lines with different EGFR expressions (A431, HT-29, HEp2, and Vero cells) and shown to accumulate within the high EGFR expressing cells up to 17-fold compared with unconjugated Pc. [83] Studies in nude mice bearing A431 and HT-29 human tumor xenografts clearly showed tumor-localized fluorescence 24 h after i.v. administration.

BODIPYs **10a,b** and **11a,b** were tested in human carcinoma HEp2 cells, overexpressing EGFR, and shown to accumulate within cells up to 90-fold compared with unconjugated BODIPY [150]. When a smaller fluorophore was used, enhanced uptake was observed for the conjugate bearing the EGFR-L2 peptide (e.g., **11b**) relative to EGFR-L1 (**11a**) [151]. This indicates that conjugation of a fluorophore to a peptide increases the hydrophobicity of the conjugate, decreasing its solubility. To increase solubility, BODIPY **12a** bearing a D-glucose moiety, or en-modified biotin, were investigated [152]. In vitro studies using SW480, HT-29, DLD-1, and LoVo cells with different EGFR overexpression showed that **12a** effectively accumulated in the high EGFR expressing cells. Furthermore, in vivo studies in HT-29 tumor-bearing mice showed tumor-localized near-IR fluorescence signal at 24 h after i.v. administration, which persisted up to 96 h, suggesting continuous uptake and slow clearance of the conjugate. More recently, to increase the in vivo stability of the fluorophore-peptide conjugate, **13a** and its derivative containing a cyclic version of EGFR-L1 were investigated in HEp2, HT-29, DLD-1, and LOVO cells [153]. These studies showed that the conjugate bearing the cyclo(K(N_3_)larllt) [84] peptide had enhanced binding affinity for the ECD of EGFR compared with **13a**, accumulating 5-fold in cells overexpressing EGFR.

We have also reported the conjugation of EGFR-L1 and EGFR-L2 to mesoporphyrin IX, via the propionic acid chains. These studies showed that conjugate **14a**, bearing two EGFR-L1 peptides, had a much higher ECD EGFR-binding affinity compared with the single peptide conjugates [149]. This might be a result of the ability of **14a** to simultaneously bind to two EGFR proteins, both in the open and closed conformations, therefore increasing its binding affinity relative to the single peptide conjugates.

The heptapeptide ATWLPPR has been the most used for targeting VEGFR. This peptide was conjugated with a tetraphenylchlorin fluorophore via a 6-aminohexanoic spacer and shown to bind to neuropilin-1 (NRP-1) recombinant chimeric protein, although not to the VEGF-2 receptor. This conjugate showed a 25-fold enhanced uptake in human umbilical vein endothelial cells (HUVEC) compared with an unconjugated fluorophore, and in vivo studies demonstrated preferential accumulation in nude mice xenografted bearing U87 human malignant gliomas [154,155,156]. The ATWLPPR peptide was also conjugated to verteporfin and investigated in a rat laser-injury model of choroidal neovascularisation (CNV) [157]. PDT treatment 1 h after the administration of the conjugate caused the complete closure of the rat lesions, while no damage was observed to the normal cells. In addition, we reported the conjugation of the ATWLPPR peptide to protoporphyrin IX via one of the propionic side chains [158]. The conjugate was observed to have 5-fold higher accumulation in human myeloid leulemia HL-60 cells versus the human carcinoma HEp2 cells, localizing preferencially in the mitochondria and lysosomes.

Several hexa- and penta-peptides devived from ATWLPPR were shown to have an affinity for binding the NRP-1 receptor, including KDKPPR, DKPRR and TKPRR. The two later pentapetides were conjugated with a tetraphenylchlorin fluorophore using three different spacers, and investigated both in vitro and in vivo. The conjugates were observed to have favorable biodistribution in an animal model, particularly the DKPPR conjugate [159].

Peptidomimetics have been investigated that effectively target the EGFR ECD inhibiting protein-protein interactions. One of such peptomimetics, designated 5-1, shown to bind to HER2 ECD and to inhibit protein-protein interactions, was conjugated with a BODIPY [160]. This conjugate accumulated in HER2-overexpressing cell lines and was observed to inhibit protein-protein interactions in vitro via a PLA assay. These peptidomimetics were also conjugated with fluorophores and doxorubicin to target the EGFR related cancer [161,162].

### 5.4. TKI Conjugates for EGFR and VEGFR Imaging

Many EGFR TKIs have been labeled with a radioactive isotope and used as EGFR or VEGFR imaging agents. The most used positron-emitting nuclides for PET imaging are ^11^C (t_1/2_ = 20.4 min), ^18^F (t_1/2_ = 110 min), and ^124^I (t_1/2_ = 20.4 mi4.2 days). For example, ^11^C-labelled erlotinib [163] and ^11^C-gefitinib [164] have been reported, as well as ^18^F-gefitinib [165] and ^18^F-afatinib [166] for in vivo imaging of tumors. Neto et al. [167] reported the synthesis and investigation of three radio-fluorinated and iodinated 4-anilinoquinazolines as potent anti-tumor imaging agents.

2-[Fluorine-18]-fluoro-2-deoxy-D-glucose (^18^F-FDG) has also been widely used for monitoring tumor treatment. Li et al. [168] demonstrated a significant correlation between EGFR-TKI treatment sensitivity in non-small cell lung cancer (NSCLC) with CD147-mediated glucose metabolic regulation using (^18^ F-FDG)-PET/CT imaging. Another potent PET tracer, N-(3-chloro-4-fluorophenyl)-7-(2-(2-(2-(2-^18^F-fluoroethoxy) ethoxy) ethoxy) ethoxy)-6-methoxyquinazolin-4-amine (^18^F-MPG) was used by Chen et al. [169] as a powerful imaging tool for quantitative detection of EGFR activating mutations.

4-Anilinoquinazoline moieties, derivatives of the TKI gefitinib, were complexed with Ru(II) via an amine side chain, and shown to have enhanced ability for causing cellular apoptosis [170]. This is not surprising, as several Ru^II^ and Ru^III^ based complexes have shown promising anticancer and antimetastasis activities [171]. The anilinoquinazoline moieties were responsible for inducing apoptosis, while the Ru^II^ center preserved the reactivity towards the DNA model compound 9-ethylguanine [170].

Bourkoula [172] and coworkers synthesized 99mTc and Re complexes with derivatized 6-amino-4-[(3-bromophenyl)amino]quinazoline to generate potent small-molecule TKIs for reversibly binding to EGFR. In vitro biological studies of both the complexes indicated their ability to inhibit EGFR phosphorylation, a key step for controlling tumor growth.

Several fluorescent Ru(II) organometallic complexes have been conjugated to 4-aminoquinazoline derivatives and used in tumor imaging and treatment [173]. Among a series of derivatives, those conjugated with 4-(3′-chloro-4′-fluoroanilino)-6-(2-(2-aminoethyl)aminoethoxy)-7-methoxyquinazoline exhibited the highest inhibitory activity against MCF-7 cancer cells, and induced enhanced cell apoptosis [173]. More recently, a fluorescent Ru(II)-bipyridine complex was conjugated with a small library of aminoquinazolines in 1:1 and 1:2 ratios [174]. The resulting conjugates accumulated in EGFR-overexpressing cells, and the most promising (4-bromophenyl)-aminoquinazoline-containing conjugate was used in fluorescence imaging of U87MG glioma cells.

**Figure 9:**
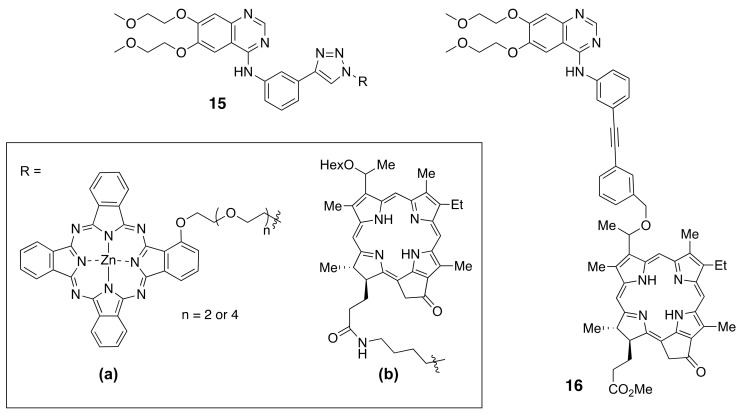
Structures of fluorophores conjugated to TKIs.

A near-IR phthalocyanine (Pc) was conjugated with erlotinib to give conjugates **15a** bearing either a tri-ethylene glycol or penta-ethyleneglycol linker [175] (Figure 9). Both conjugates were highly phototoxic to HEpG2 cells with EGFR-overexpression, and there was no observed influence on the size of the oligoethylene glycol linker. Fluorescence imaging of nude mice bearing A431 tumors showed 5-fold higher tumor-localized fluorescence using **15a** compared with fluorophore alone.

Afatinib has been conjugated with cyanine-based fluorophores (Cy3 and Cy5) and the resulting conjugates shown to be efficient theranostics agents for HER1 and HER2-overexpressing tumor cells and for imaging A549 xenograft animal tumors [176]. A quinazoline derivative has also been conjugated with a 5(6)-carboxytetramethylrhodamine fluorophore and shown to have an efficiency of 34% for labeling EGFR [177].

Recently, Pandey and coworkers [178,179] reported the conjugation of pheophorbide a derivatives to erlotinib at different positions around the ring, including the synthesis of **15b** and **16** (Figure 9). A series of near-IR conjugates were obtained and investigated, including derivatives of pyropheophorbide, λ_max_ ~ 600 nm, purpurinimide, λ_max_ ~ 700 nm, bacteriopupurinimide, λ_max_ ~ 782 nm, and chlorin e_6_, λ_max_ ~ 660 nm were conjugated with erlotinib using various linkers. The conjugates were evaluated in cellular uptake, cytotoxicity, and in SCID-tumor bearing mice studies. A radioactive analog of conjugate **15b** bearing ^124^I was shown to be a promising dual agent for imaging (PET, fluorescence) and phototherapy (PDT) of bladder tumors. However, the most promising conjugate based on its higher tumor cell accumulation and in vivo tumor control was found to be **16**. This conjugate had the highest uptake into SCID mice bearing either UMUC3 bladder or FaDu head and neck tumors and faster clearance from the liver and normal tissues compared with the photosensitizer alone and other conjugates with erlotinib at different points of attachment. Conjugate **16** also showed improved long-term tumor control compared with the photosensitizer alone at a similar administered dose and light PDT treatment parameters.

## 6. Conclusions

EGFR and VEGFR are important targets for cancer diagnosis and treatment. A large number of ligands have been developed that target these families of receptors, from high molecular weight antibodies, affibodies and nanobodies, to low molecular weight TKIs and small peptides. The targeting of these receptors has allowed for efficient imaging and targeted therapy options for various tumors that overexpress these proteins, advancing the field of focused and personalized medicine of cancer. Research in this field requires the interdisciplinary work of chemists, radiologists, and biologists to achieve the conjugation of an impressive variety of imaging agents, ranging from fluorophores to radioisotopes and QDs, to various antiEGFR, antiVEGFR, TKIs, and peptides. While antibody-based molecules have high affinity and specificity to bind to EGFR, small molecule TKI and peptides have lower cost synthesis, lower immunogenicity, faster clearance, better intratumoral diffusion, and easier functionalization with imaging probes compared with antibodies, while showing high specificity for EGFR-targeting. The structures of the ligands, the nature of the imaging agent, the modes of attachment, and linkers used all determine the tumor specificity and therapeutic efficacy of the resulting conjugates. As more structural details of EGFR and VEGFR with their target peptides and natural ligands are elucidated, new generations of stable peptide molecules can be used for conjugation with imaging probes, particularly with pyrrole-based fluorophores, and used for tumor detection, diagnostic imaging, and cancer treatment.

## Figures and Tables

**Table 1:** EGFR Ligands and their Classification.

**High-Affinity Ligands**	1. Epidermal growth factor (EGF)
	2. Transforming growth factor alpha (TGF-a)
	3. Heparin-binding EGF-like growth factor (HB-EGF)
**Low-Affinity Ligands**	4. Amphiregulin (AREG)
	5. Epiregulin (EREG)
	6. Epigen (EPGN)
	7. Betacellulin (BTC)
**Neuregulins**	8. Neuregulin-1 (NRG1)
	9. Neuregulin-2 (NRG2)
	10. Neuregulin-3 (NRG3)
	11. Neuregulin-4 (NRG4)

**Table 2:** VEGF ligands and their receptor binding partner(s).

Ligand	Known VEGFR Receptor Binding
VEGF-A	VEGFR-1, VEGFR-2 (angiogenesis)
VEGF-B, PlGF	VEGFR-1 (angiogenesis)
VEGF-C, VEGF-D	VEGFR-2 (Angiogenesis), VEGFR-3 (lymphangiogenesis)
VEGF-E	VEGFR-2 (angiogenesis)
VEGF-F [39]	VEGFR-1, VEGFR-2 (angiogenesis)

**Table 3:** Imaging modalities and contrast agents are commonly used for EGFR and VEGFR families of receptors.

Imaging Modality	Targeted Receptor	Imaging Agent	Therapeutic Agent/Targeting Moiety	Application
SPECT	ErbB2	111In [99]	111In-DTPA-SV2-61r	Adenocarcinoma
SPECT	ErbB2	99mTc [100]	99mTc-ICR12	Breast cancer
SPECT	ErbB2	99mTc [101]	99mTc-CIBCgp185	Breast cancer
PET	ErbB2	124I [102]	124I-ICR12	Breast cancer
SPECT	ErbB2	131I [103]	131I-herceptin	Mammary adenocarcinoma
PET	ErbB2	186Re [104]	186Re-labeled 4D5 186Re-labeled-rhuMAb HER2	
PET	ErbB2	177Lu [105]	177Lu-isothiocyanate-benzyl-CHX-A”-DTP Apertuzumab	
MicroPET + MRI	ErbB2	(86)Y [106]	(86)Y-labeled trastuzumab	Ovarian cancer
MRI	ErbB2	Superparamagnetic iron oxide (SPIO) particles [107]	Streptavidin-conjugated superparamagnetic nanoparticles - transtuzumab	Breast cancer
MRI	ErbB2	Superparamagnetic iron oxide (SPIO) particles [108]	Herceptin-iron oxide nanoparticles	Breast cancer
SPECT	ErbB2	99mTc [109]	[(99m)Tc]-HYNIC-trastuzumab Fab	Breast cancer
SPECT	ErbB2	111In [110]	111In-DTPA-Trastuzumab Fab	Breast cancer
US	VEGFR2	Microbubbles [111]	MBKDR	Colon cancer
MRI	EGFR	Magnetic iron-oxide nanoparticles (IONP) [112]	Cetuximab-IONP	Brain tumor
MRI	EGFRvIII	Magnetic iron-oxide nanoparticles (IONP) [113]	EGFRvIIIAb-IONP	Glioblastoma
MRI	EGFRvIII	Superparamagnetic iron oxide nanoparticles (SPIONs) [114]	PEPHC1-SPIONs	Glioblastoma
MRI	EGFR	SPIONs [115]	EGFRmAb-SPIONs	Glioblastoma
MRI	EGFR	SPIONs [116]	SPION-EGF	Glioblastoma
Optical Imaging	ErbB2	Quantum dots (QDs) [117]	QD-IgG	Breast cancer
Optical Imaging	ErbB2	Nano shells [118]	Anti-IgG-PEG-Nano shells	Breast cancer
Optical imaging (bioluminescence)	EGFR	Gelatin nanoparticles (NPs) [119]	Gemcitabine-gelatin NPs	Pancreatic cancer
Optical imaging	EGFR	Gold nanoparticles (AuNPs) [120]	C225-AuNPs	Non-small cell lung cancer (NSCLC)
Optical imaging	HER2	Gold nanorods (GNRs) [121]	Her-PEG GNRs	Breast cancer
Optical imaging (Near IR)	EGFR	Gold nanorods (GNRs) [122]	Cetuximab-GNRs	Squamous cell carcinoma (SCC)
Optical imaging (Near IR)	EGFR	Gold nanorods (GNRs) [123]	CO-GNRs	Oral adenosquamous carcinoma
